# Sirolimus-eluting balloon for the treatment of peripheral artery disease: SUCCESS PTA real-world single-arm study

**DOI:** 10.1186/s42155-026-00751-2

**Published:** 2026-08-08

**Authors:** Michael Lichtenberg, Thomas Zeller, Ashish Patel, Ralph Kickuth, Julio Andrés Pascua, Jörg Schmehl, Klaus Hertting, Konstantinos Katsanos, Ralph Oberacker, Aljoscha Rastan, Stefan Müller-Hülsbeck, Hans-Martin Gissler, Grigorios Korosoglou, Christian Heiss, Christos Rammos

**Affiliations:** 1 Arnsberg Vascular Center, Alexianer Klinikum Hochsauerland, Stolte Ley 5, Arnsberg, 59759 Germany; 2 Department of Cardiology and Angiology, Medical Center, University of Freiburg, Bad-Krozingen, Germany; 3 Academic Department of Vascular Surgery, Guy’s & St Thomas’ NHS Foundation Trust and King’s College London, London, UK; 4 Department of Diagnostic and Interventional Radiology, University Hospital Würzburg, Würzburg, Germany; 5 Departamento Hemoterapia y Cardiologia Intervencionista, Hospital Italiano la Plata, La Plata, Buenos Aires Argentina; 6 Diagnostic and Interventional Radiology, University Hospital of Tübingen, Tübingen, Germany; 7 Department of Cardiology and Angiology, Krankenhaeuser Buchholz und Winsen gGmbH, Buchholz in der Nordheide, Germany; 8 Vascular & Interventional Radiology School of Medicine, University of Patras Rion, Patras, Greece; 9 Chefarzt Innere Medizin SRH Klinikum Karlsbad-Langensteinbach GmbH, Karlsbad, Germany; 10 Department of Angiology, Lucerne Cantonal Hospital, University Teaching and Research Hospital, Lucerne, Switzerland; 11 Academic Hospitals Flensburg of Christian-Albrechts-University Kiel – Faculty of Medicine, Deaconess Hospital gGmbH, Flensburg, Germany; 12 Interventional Radiology Kantonsspital Aarau, Aarau, Switzerland; 13 Department of Cardiology and Angiology, GRN-Hospital Weinheim, Weinheim, Germany; 14 Department of Cardiology and Angiology, GRN-Hospital Eberbach, Eberbach, Germany; 15 Department of Clinical and Experimental Medicine, Faculty of Health and Medical Sciences, University of Surrey, Guildford, GU2 7XH UK; 16 Vascular Medicine Department, Surrey and Sussex Healthcare NHS Trust, Redhill, RH1 5RH UK; 17 Department of Cardiology and Vascular Medicine, West German Heart and Vascular Center, University of Duisburg Essen, Essen, Germany

**Keywords:** Peripheral artery disease, Endovascular therapy, Sirolimus-eluting balloon

## Abstract

**Background:**

Outcomes with sirolimus-coated drug-eluting balloons (DEB) have been positive for the treatment of femoropopliteal arterial lesions but have not been studied in a broader patient population.

**Objectives:**

Collect real-world safety and effectiveness data on the SELUTION SLR DEB in patients with claudication and chronic limb-threatening ischemia (CLTI).

**Methods:**

This international multi-center, prospective, single-arm, post-market study (SUCCESS PTA) enrolled 723 patients at 27 sites. The primary endpoint was freedom from clinically driven target lesion revascularization (CD-TLR) after 1 year.

**Results:**

Median age was 71.0 [63.0–79.0] years, 65% were male, 25.7% had CLTI. In total, 822 lesions were included; median lesion length was 100.0 mm [50.0–190.0] with 42.1% chronic total occlusions and 36.4% moderate-to-severe calcification. At 12 months, freedom from CD-TLR was 91.1%, 95% CI [88.6–93.1] and 52.1% of patients were asymptomatic. Median EQ-VAS score and ankle-brachial pressure index improved significantly from baseline to 12 months (65.0 [50.0–80.0] vs. 75.0 [60.0–85.0] (*p* < 0.0001)) and (0.6 [0.5–0.8] vs. 0.9 [0.8–1.0] (*p* < 0.0001)), respectively.

At 12 months, outcomes in CLTI patients included major target limb amputation, 5.8%; major cardiac event rate, 4.3%; and death, 11.3%. Respective outcomes for those with claudication were 0.0%, 2.2% and 1.2%.

**Conclusions:**

This real-world single-arm study is the largest assessment of a sirolimus DEB for treatment in claudication and CLTI. The outcomes were consistent with those reported for paclitaxel-coated balloons and support this DEB platform as a promising alternative, particularly in CLTI patients.

**Level of evidence:**

Level 3.

**Supplementary Information:**

The online version contains supplementary material available at https://doi.org/10.1186/s42155-026-00751-2.

## Introduction

Drug-coated balloons (DCBs) have become a standard of care for the treatment of femoropopliteal disease because of their superior primary patency and target lesion revascularization rates compared to non-coated balloon angioplasty [1–8]. However, results from the SWEDEPAD 1 and SWEDEPAD 2 randomized controlled trials showed that paclitaxel-coated balloons fail to improve disease-specific quality of life in patients with claudication and amputation rates in CLTI patients [9, 10]. This has led to a shift in ESVS guideline recommendations of paclitaxel-coated balloons for asymptomatic peripheral artery disease (PAD) and intermittent claudication. As of 2026, ESVS recommends against routine use of paclitaxel-coated balloons for intermittent claudicant, citing a lack of quality of life benefit and the potential for long-term mortality risks [11]. In addition, studies on paclitaxel-coated balloons in below-the-knee (BTK) lesions have shown mixed positive and negative results, making their superiority over plain old balloon angioplasty (POBA) unclear [12–17].

A promising alternative to paclitaxel, which is a cytotoxic drug, may be represented by sirolimus. Sirolimus acts cytostatically to inhibit cell proliferation by reversibly putting the cells into the resting phase of mitosis (G_0_). Sirolimus also has a larger local margin of safety, wider therapeutic window and anti-inflammatory effects [18]. Until recently, sirolimus could not be delivered on an angioplasty balloon platform because it is less lipophilic than paclitaxel, making its transfer and absorption into the vessel wall cumbersome. The SELUTION Sustained Limus Release (SLR™) Drug-Eluting Balloon (DEB, MedAlliance LLC (a Cordis Company), Irvine, USA) circumvents this limitation by using micro-reservoirs, a mix of sirolimus and biodegradable poly lactic-co-glycolic acid (PLGA), applied to the balloon surface using a proprietary blend of lipid-based excipients. This coating controls drug release and optimizes sirolimus transfer and adherence to the vessel wall using a nominal dose density (1 μg/mm^2^) on the balloon surface. Upon balloon expansion, these micro-reservoirs are transferred to the tissue and maximize delivery of drug to the surrounding cells. Sirolimus is eluted and absorbed slowly, with studies in coronary porcine models sustaining release up to 90 days [19, 20].

Early studies of the SELUTION SLR SEB have demonstrated high primary patency rates (typically > 85%), low target lesion revascularization rates (< 10%), and encouraging safety profiles across femoropopliteal and tibial disease [20–28]. Similarly, promising results have been shown of other limus-eluting devices within the SIRONA trial, which demonstrated non-inferiority to paclitaxel balloons, and in SirPAD, that demonstrated superiority to percutaneous transluminal angioplasty (PTA) in a mixed ATK/BTK setting [29, 30]. However, these studies have been limited by modest sample sizes, restricted lesion lengths, and selective inclusion criteria, often excluding patients with advanced disease or complex anatomy. The SUCCESS PTA study was designed to evaluate the performance of SELUTION SLR DEB in a broader and more clinically representative population in an international real-world setting. This post-market, all-comers single-arm study provides one of the largest assessments to date of sirolimus-eluting balloon angioplasty in peripheral arterial disease.

## Methods

### Study design

SUCCESS PTA Study is an open-label, real-world all-comers, prospective, multi-center, single-arm study. It enrolled patients from 27 centers in eight countries. The study protocol was approved by the ethics committee at each site, and the trial was registered with clinicaltrials.gov (NCT04776434). The trial was conducted in compliance with good clinical practice for medical devices and in accordance with ethical principles based on the Declaration of Helsinki.

### Patient population

Patients undergoing treatment for peripheral arterial disease were eligible for this trial if they were ≥ 18 years of age and deemed by the operator suitable for treatment with the SELUTION SLR DEB. Patients with life expectancy of less than 12 months or those deemed, in the investigator’s judgment, unlikely to adhere to study follow-up were excluded.

### Study device

The SELUTION SLR DEB is a 3.9 Fr (1.3 mm) diameter over-the-wire device compatible with a 0.018–inch guidewire, delivered via a 5–7 French access sheath depending on device size. The DEB diameters range from 2.0 to 7.0 mm with lengths of 20–150 mm.

### Study procedure

Patients were treated per standard of care at each institution. Pre-dilation or vessel preparation such as atherectomy, thrombectomy and scoring balloons were performed in all cases. For this registry, lesion preparation was performed at the operator’s discretion with no restrictions, based on local practice and standard of care. A post-angioplasty angiogram was captured to document procedural outcomes. Lesion calcification was assessed per the PACCS classification [31]. In instances of residual stenosis > 30% or a flow-limiting dissection, investigators were permitted to follow standard-of-care bailout procedures at their discretion such as repeat PTA, repeat SELUTION SLR DEB and/or placement of a bail-out stent. Post-procedure dual anti-platelet treatment was prescribed per each institution’s standard of care.

### Outcomes and follow-up

Resting ankle brachial pressure index (ABPI), Rutherford classification, EQ-5D and EQ-VAS data were collected at pre-procedure (within 30 days prior), 6 months, and 12 months post-procedure. Results are based on paired data and include only subjects with complete data at the relevant timepoints, and follow-up completed within the ± 30-day visit window. Adverse events, hospital resource utilization data and concomitant medications were also collected through 12 months. All events were assessed by the Cordis Sponsor Safety Office: site-reported information entered in the EDC system was reviewed to categorize the event (determine relevance to each study endpoint) and evaluate causal relatedness.

The primary endpoint was freedom from clinically driven target lesion revascularization (CD-TLR). Secondary endpoints included device success defined as successful delivery, balloon inflation, deflation, and retrieval of the intact device without burst below rated burst pressure; procedure success defined as device success with a final target lesion residual stenosis ≤ 50% at the end of the procedure with or without bail-out stenting. Additional secondary endpoints included EQ-5D and a change in Rutherford classification, ABPI, and EQ-VAS from baseline and any major adverse limb event (MALE), defined by the composite of severe limb ischemia leading to an intervention or major amputation (above the ankle).

Time to event analyses were conducted using the Kaplan–Meier method. Categorical variables are presented as frequencies and percentages, while continuous variables are summarized with median and inter-quartile range (IQR) as the p-value associated with the Shapiro–Wilk test to assess normality was < 0.05 for all continuous variables presented. Event proportions were calculated based on the number of eligible patients who completed follow-up or experienced the event of interest prior to follow-up and had complete data for the variable of interest. Signed-rank tests were used to test if the change from baseline to a follow-up time point was significant for ABPI and EQ-VAS. For Rutherford classification, the ordinal McNemar-Bowker test was used to test if the change from baseline to a follow-up time point was significant. Data was analyzed using Stata software (version 18.5, StataCorp College Station, Texas, USA).

## Results

### Study population and baseline characteristics

**Fig. 1:**
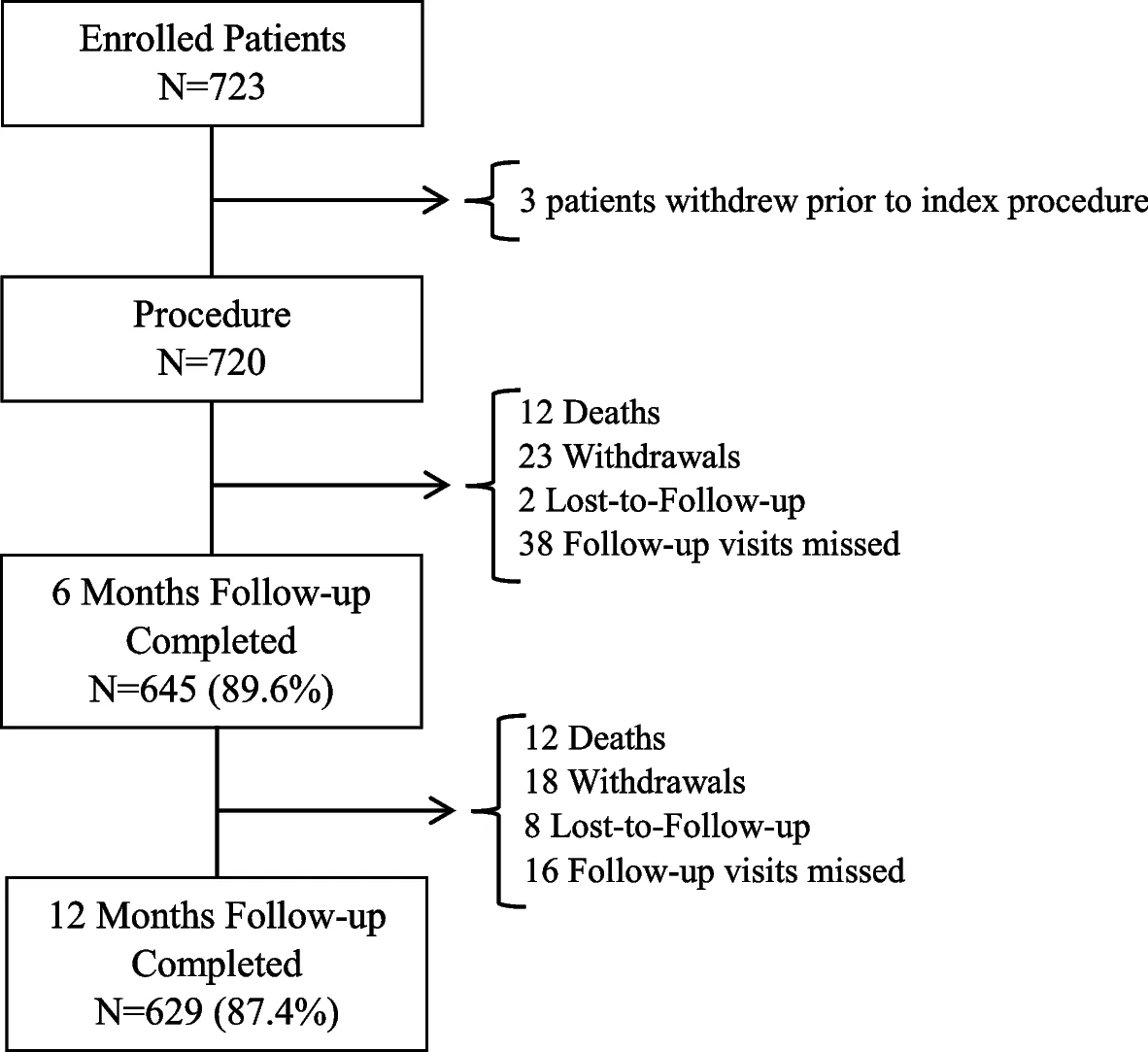
Patient flow diagram through 365 days (± 30 days)

Between February 2021 and October 2023, 723 patients were enrolled at 27 sites (Supplemental Table 1). Of these, three withdrew consent and declined the use of their previously collected data, resulting in a final analysis population of 720 patients (Fig. 1). Baseline patient and lesion characteristics are shown in Table 1. Overall, the median age was 71.0 [63.0–79.0], 65.0% (468/720) of patients were male, 37.9% (273/720) had diabetes and 30.3% (217/715) were current smokers. Patient characteristics in those with claudication (*n* = 534) versus CLTI (*n* = 186) are listed in Table 1.

**Table 1:** Baseline patient characteristics

	Full cohort (*N* = 720 patients)	Claudicants (*N* = 534 patients)	CLTI (*N* = 186 patients)
Age (years)	71.0 [63.0–79.0]	71.0 [63.0–77.0]	74.0 [64.0–84.0]
Male	65.0% (468/720)	63.9% (341/534)	68.3% (127/186)
Diabetes mellitus	37.9% (273/720)	33.0% (176/534)	52.2% (97/186)
Insulin dependent	13.6% (98/720)	9.4% (50/534)	25.8% (48/186)
Previous intervention (< 1 year) at lesion	16.6% (119/718)	14.8% (79/532)	21.5% (40/186)
Hypertension	85.3% (614/720)	84.5% (451/534)	87.6% (163/186)
Hyperlipidemia	76.8% (553/720)	77.0% (411/534)	76.3% (142/186)
Cerebral stroke/transient ischemic attack	7.1% (51/720)	5.6% (30/534)	11.3% (21/186)
Myocardial infarction	14.9% (107/720)	15.0% (80/534)	14.5% (27/186)
Renal failure/impairment	16.8% (121/720)	12.7% (68/534)	28.5% (53/186)
Current smoker	30.3% (217/715)	31.8% (168/529)	26.3% (49/186)
Rutherford Categories (RC)			
1	3.2%	4.3%*	NA
2	21.4%	28.8%	NA
3	49.4%	66.7%	NA
4	8%	NA	31.2%
5	16.5%	NA	64%
6	1.2%	NA	4.8%
Ankle-Brachial Pressure Index (ABPI)	0.6 [0.5, 0.8] (598)	0.7 [0.5, 0.8] (463)	0.6 [0.4, 0.8] (135)

Data are presented as percentages % (*n*/*N*) or median [IQR]. Values represent total n unless otherwise specified

^*^There were 23 patients (27 lesions) classified as Rutherford 1, 85% of lesions were de novo, 78% had ≥ 80% stenosis, all had more than 50% stenosis and were enrolled per investigator discretion. NA for not applicable

Most lesions were de novo (75.1%) with a median lesion length of 100.0 [50.0–190.0] mm; 42.1% were chronic total occlusions and 36.4% were moderately to severely calcified. Baseline lesion characteristics are provided in Table 2.

**Table 2:** Baseline lesion characteristics

	Full cohort (*N* = 822 lesions)	Claudicants (*N* = 604 lesions)	CLTI (*N* = 218 lesions)
Reference vessel diameter (mm)	5.0 [5.0–6.0] (821)	5.2 [5.0–6.0]	5.0 [4.0–6.0] (217)
Lesion length (mm)	100.0 [50.0–190.0] (818)	100.0 [45.0–200.0] (601)	100.0 [50.0–180.0] (217)
De novo lesion	75.1% (617/822)	75.2% (554/604)	74.8% (163/218)
Restenotic lesion˚	24.9% (205/822)	24.8% (150/604)	25.2% (55/218)
Diameter stenosis (%)	98.0 [90.0–100.0]	95.0 [90.0–100.0]	99.0 [90.0–100.0]
Total occlusion	42.1% (346/822)	41.7% (252/604)	43.1% (94/218)
Located above-the-knee*	81.0% (666/822)	85.9% (519/604)	67.4% (147/218)
SFA prox	38.3% (255/666)	37.8% (196/519)	40.1% (59/147)
SFA mid	50.2% (334/666)	52.4% (272/519)	42.2% (62/147)
SFA dis	52.0% (346/666)	48.7% (253/519)	63.3% (93/147)
POP 1	30.3% (202/666)	26.6% (138/519)	43.5% (64/147)
POP 2	21.8% (145/666)	19.3% (100/519)	30.6% (45/147)
Other**	2.9% (19/666)	2.9% (15/519)	2.7% (4/147)
Located below-the-knee*	19.0% (156/822)	14.1% (85/604)	32.6% (71/218)
POP 3	42.3% (66/156)	54.1% (46/85)	28.2% (20/71)
Anterior tibial	23.7% (37/156)	14.1% (12/85)	35.2% (25/71)
Posterior tibial	9.6% (15/156)	5.9% (5/85)	14.1% (10/71)
TP trunk	9.6% (15/156)	5.9% (5/85)	14.1% (10/71)
Peroneal	5.1% (8/156)	3.5% (3/85)	7.0% (5/71)
Other**	7.7% (12/156)	9.4% (8/85)	5.6% (4/71)
Median lumen diameter (mm)	0.5 [0.0–1.0] (816)	0.5 [0.0–1.0] (599)	0.1 [0.0–1.0] (217)
PACCS calcification grade 3†	20.1% (165/821)	19.9% (120/603)	20.6% (45/218)
PACCS calcification grade 4†	16.3% (134/821)	15.6% (94/603)	18.3% (40/218)

Data are presented as percentages % (*n*/*N*) or median [IQR]. Values represent total n unless otherwise specified

˚Includes in-stent restenosis and native vessels

^*^One lesion can cross multiple segments; above-the-knee: proximal target lesion origin of Pop 2 and above (including common femoral), below-the-knee: any lesion including segments below POP2, BTK lesions either isolated or combined with inflow (ATK) lesions

^**^For ATK: Profunda, iliac, bypass; For BTK: bypass, dorsalis, plantar

^†^Peripheral Arterial Calcium Scoring System (PACCS) Grade 3: bilateral calcification < 5 cm, Grade 4: bilateral calcification ≥ 5 cm

### Interventional procedure

Lesion preparation was performed in 98.8% of lesions, most commonly with standard balloon angioplasty (84.1%). Adjunctive techniques included atherectomy (22.2%), scoring balloons (4.1%), intravascular lithotripsy (3.8%) and thrombectomy (3.3%). Bail-out stenting occurred in 32.0% of the cases. The bail-out lesions had a median length of 150 mm. Additional procedure details are provided in Table 3. Device success was achieved in 99.0% of cases and procedure success was noticed in 97.9% of patients. 13 patients did not achieve device success (9 issues with deployment, 2 issues with inflation/deflation and 2 ruptures).

**Table 3:** Procedural characteristics

	Full cohort (*N* = 822 lesions)	Claudicants (*N* = 604 lesions)	CLTI (*N* = 218 lesions)
Lesion preparation	98.8% (809/819)	98.5% (593/602)	99.5% (216/217)
Device type for lesion preparation			
POBA*	84.1% (677/805)	84.9% (502/591)	81.8% (175/214)
Atherectomy**	22.2% (179/805)	22.0% (130/591)	22.9% (49/214)
Thrombectomy	3.3% (27/805)	2.9% (17/591)	4.7% (10/214)
Scoring balloon	4.1% (33/805)	3.2% (19/591)	6.5% (14/214)
IVL*	3.8% (31/805)	3.7% (22/591)	4.2% (9/214)
Cutting balloon	1.7% (15/805)	1.9% (11/591)	1.9% (4/214)
Bail-out stenting˚	32.0% (263/822)	33.8% (204/604)	27.1% (59/218)
Reason (could be more than 1)			
Residual stenosis > 30%	50.9% (134/263)	52% (106/204)	45.8% (27/59)
Flow limiting dissection	48.3% (127/263)	50% (102/204)	52.5% (31/59)
Other^†^	6.5% (17/263)	4.9% (10/204)	11.9% (7/59)
Final residual stenosis (%)	10.0 [0.0–20.0] (821)	10.0 [0.0–20.0] (603)	10.0 [0.0–15.0] (218)
Device success	99.0% (1296/1309^‡^)	99.2% (940/948^‡^)	98.6% (356/361^‡^)
Procedure success	97.9% (705/720)	98.3% (525/534)	96.8% (180/186)

Data are presented as percentages % (*n*/*N*) or median [IQR]. Values represent total n unless otherwise specified

^*^POBA plain old balloon angioplasty, *IVL* intravascular lithotripsy

^**^Type of atherectomy included 140 rotational, 20 directional, 7 orbital, and 12 were not reported

˚Median lesion length of lesions with bail-out stenting was 150 mm

^†^Other reasons included calcification [2], recoil [11], and thrombus [4]

^‡^Total number of devices

Device Success: Successful delivery, balloon inflation, deflation and retrieval of the intact study device without burst below rated burst pressure

Procedure Success: Device success and residual stenosis ≤ 50% at the end of the procedure

### Clinical outcomes

**Fig. 2:**
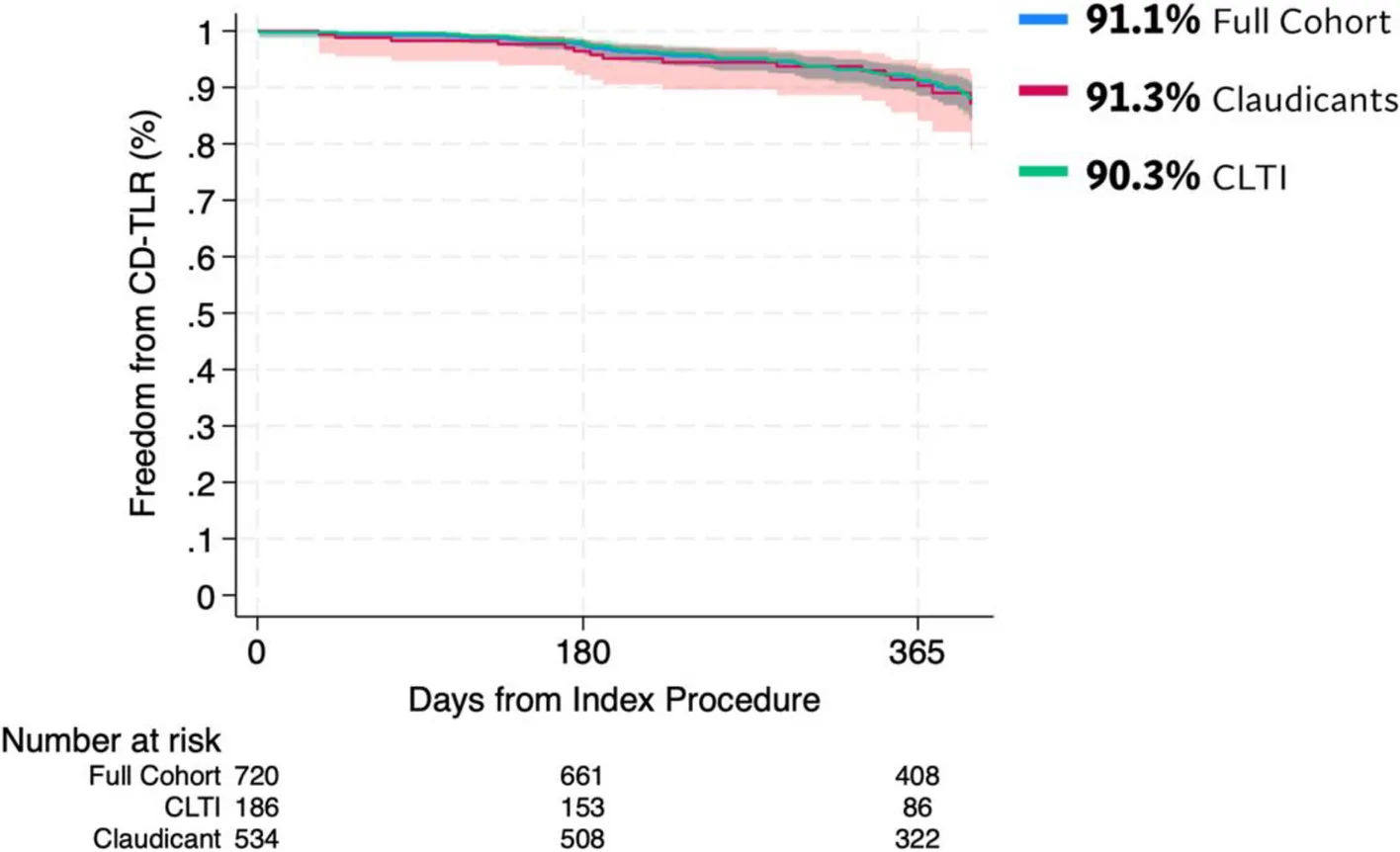
Freedom from clinically driven target lesion revascularization through 365 days

In total, 645 and 629 patients completed follow-up at 6 months and 12 months, respectively. Freedom from CD-TLR was 97.5%, 95% CI [96.1, 98.5] at 6 months and 91.1%, 95% CI [88.6–93.1] at 12 months for the full cohort (Fig. 2). At 12 months, freedom from CD-TLR was 91.3% 95% CI [88.5, 93.5] for claudicants and 90.3%, 95% CI [84.0, 94.2] for CLTI patients. The cumulative MALE rate for the full cohort was 5.9% and 13.2% at 6 and 12 months, respectively. In the full cohort, major target limb amputation rates were 1.1% at 6 months and 1.3% at 12 months, while no amputations were performed in the claudicant group and eight major target limb amputations (5.8%) were performed in the CLTI group. Death rates were 1.8% at 6 months and 3.5% within 12 months of the procedure for the full cohort, 1.2% in claudicants and 11.3% in CLTI patients at 12 months. Clinical outcomes are summarized in Table 4.

**Table 4:** Clinical outcomes at 6 and 12 months

Outcomes	Full Cohort		Claudicants		CLTI	
	6 Months	12 Months	6 Months	12 Months	6 Months	12 Months
Death	1.8% (12/657)	3.5% (23/652)	0.2% (1/501)	1.2% (6/501)	7.1% (11/156)	11.3% (17/151)
Major cardiac event^1^	1.1% (7/649)	2.7% (17/636)	0.8% (4/501)	2.2% (11/497)	2.0% (3/148)	4.3% (6/139)
Clinical driven target lesion revascularization	2.6% (17/646)	9.0% (57/631)	2.2% (11/501)	8.7% (43/496)	4.1% (6/145)	10.4% (14/135)
Thrombosis	0.8% (5/647)	0.8% (5/629)	0.4% (2/500)	0.4% (2/495)	2.0% (3/147)	2.2% (3/134)
Target limb amputation	1.9% (12/647)	2.1% (13/634)	0.0% (0/500)	0.0% (0/496)	8.2% (12/147)	9.4% (13/138)
Major target limb amputation	1.1% (7/646)	1.3% (8/633)	0.0% (0/500)	0.0% (0/496)	4.8% (7/146)	5.8% (8/137)
Rutherford Class 4		12.5% (1/8)				
Rutherford Class 5		50% (4/8)				
Rutherford Class 6		37.5% (3/8)				
Minor target limb amputation	0.8% (5/646)	0.8% (5/630)	0.0% (0/500)	0.0% (0/495)	3.4% (5/146)	3.7% (5/135)
MALE^2^	5.9% (38/648)	13.2% (84/638)	4.2% (21/501)	11.4% (57/498)	11.6% (17/147)	19.3% (27/140)

Denominators reflect the number of patients with available data for each variable; totals may vary due to missing or incomplete data

^1^Major Cardiac Event: myocardial infarction, stroke, cardiovascular death

^2^Major Adverse Limb Event (MALE)-severe limb ischemia leading to an intervention to target limb or major vascular amputation

**Fig. 3:**
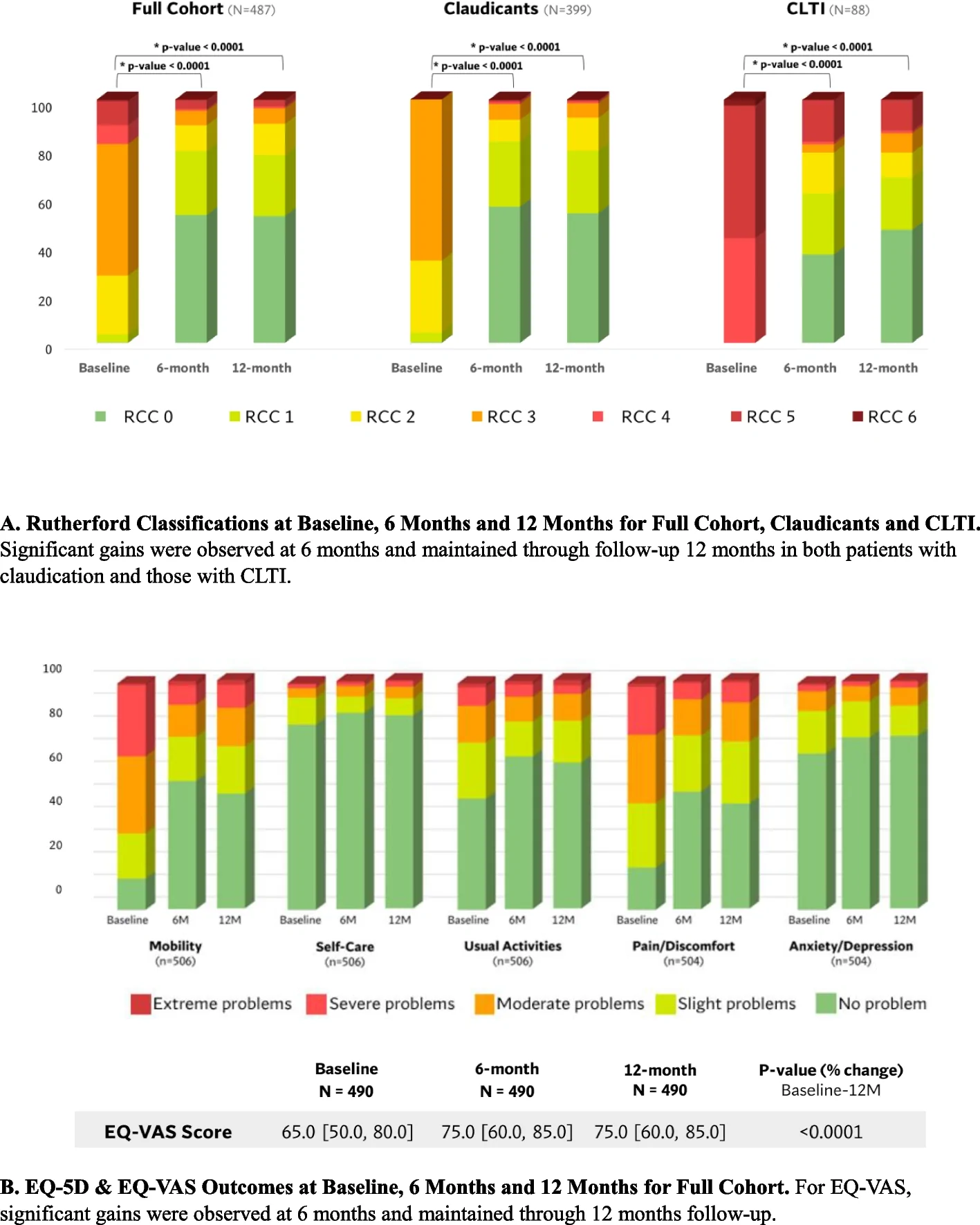
Rutherford classification, EQ-5D and EQ-VAS outcomes

A paired analysis of the full cohort showed that Rutherford categories (RC) significantly improved at 6 months compared with baseline values and improvements were sustained after 12 months (*p* < 0.0001, vs baseline). In total, 52.2% were asymptomatic at 12 months and 88.1% improved by at least one RC from baseline (Fig. 3).

The full cohort median EQ-VAS score improved significantly from 65.0 [50.0–80.0] at baseline to 75.0 [60.0–85.0] after 6 months (*p* < 0.0001) and was sustained after 12 months 75.0 [60.0–85.0] (*p* < 0.0001, Fig. 3). A paired analysis of EQ-VAS data (*n* = 405) for patients with claudication at baseline, 6 months, and 12 months showed an improvement from 65.0 [50.0, 80.0] to 75.0 [60.0, 85.0] and 75.0 [60.0, 85.0] (*p* < 0.0001). The paired analysis of EQ-VAS data (*n* = 93) at baseline, 6 months, and 12 months in CLTI patients showed improvement from baseline 60.0 [50.0, 70.0] to 70.0 [50.0, 80.0] at 6 months (*p* < 0.0001) and then 65.0 [50.0, 80.0] at 12 months (*p* = 0.0132).

Likewise, the resting ABPI improved significantly in the full cohort from baseline (06 [0.5–0.8]) to 0.9 [0.8, 1.0] at 6 months and remained high (0.9 [0.8–1.0]) after 12 months (*p*-value < 0.0001 vs. baseline). ABPI data in patients with claudication at baseline, 6 months and 12 months (*n* = 307) showed a median baseline of 0.7 [0.5, 0.8], improving to 0.9 [0.8, 1.0] at 6 months and 0.9 [0.8, 1.1] at 12 months (*p* < 0.0001). A paired analysis of ABPI in CLTI patients (*n* = 59) showed a baseline median of 0.6 [0.4, 0.8] improving to 0.9 [0.8, 1.0] at 6 months and 0.9 [0.8, 1.0] at 12 months (*p* < 0.0001).

## Discussion

The SUCCESS PTA study demonstrated an overall CD-TLR rate of 9.0% at 1 year. These real-world outcomes are encouraging for the adoption of sirolimus-eluting balloons considering the inclusion of CTLI patients and below-the-knee disease. In a Delphi consensus for definitive treatment strategies of femoropopliteal lesions, vascular specialists from different countries and disciplines showed high agreement rates for the use of DCBs, including sirolimus-coated balloons [32]. Recently, SIRONA, a head-to-head randomized controlled, noninferiority study of a sirolimus- and paclitaxel-coated balloon in femoropopliteal disease, demonstrated noninferiority of sirolimus to paclitaxel in primary patency and composite freedom from CD-TLR, major target limb amputation and device- or procedure-related death [30]. The SirPAD randomized, controlled non-inferiority trial compared a sirolimus-coated balloon to uncoated-balloon in infrainguinal artery disease. The SirPAD trial demonstrated noninferiority and subsequent superiority with regard to major adverse limb events at 1 year [29]. These RCTs, along with real-world data from SUCCESS, suggest that sirolimus-coated balloons are a viable tool in the treatment of PAD.

Early studies evaluating the SELUTION SLR SEB demonstrated favorable outcomes in patients with femoropopliteal disease, but were generally limited by small sample sizes, less complex lesions, and predominantly claudicant populations. The first-in-human study reported a freedom from TLR rate at 98% at 6 months in relatively short lesions and limited chronic total occlusions [28]. The SELUTION SFA Japan study enrolled a larger cohort of claudicants with more complex femoropopliteal disease including longer lesion lengths and a high prevalence of severe calcification. The 12-month patency rate was 87.9% and TLR-free rate of 97.0% [24].

As stated previously, until this current study, the SELUTION SLR DEB has been mainly studied in claudicant populations with primarily SFA disease. More recently, small single-center registries have examined real-world use of the SELUTION SLR SEB in broader patient populations and lesion presentations such as CLTI and BTK disease. These studies have reported encouraging patency and TLR outcomes despite challenging lesion characteristics and low rates of bailout stenting [21, 23, 25]. The 91.1% freedom from CD-TLR through 12 months observed in the present study is in-line with prior studies, validating early promising results in a broader and larger population. Despite 98.9% lesion vessel preparation reported in the study, the majority was conducted with POBA. The bail-out stent rate was 32.0% in this study, which is not surprising given the median lesion length was 150 mm compared to 100 mm in the overall cohort. 42.1% of lesions reported CTOs and advanced vessel preparation techniques were not widely used. High bailout rates likely reflect lesion complexity rather than failure of the sirolimus technology itself. The benefits of advanced vessel preparation techniques in combination with a sirolimus-coated DEB remain an area of potential future exploration.

One of the benefits of the SUCCESS PTA study as an all-comers registry was that inclusion criteria allowed patients with a wide degree of symptomology and lesion complexity, including both (71.5%) claudicant and (28.5%) CLTI patients, as well as (81.0%) ATK and (19.0%) BTK lesions. The SUCCESS PTA study demonstrated an overall CD-TLR rate of 9.0% at 1 year. When outcomes were stratified by severity of symptoms at baseline, patients with claudication had a CD-TLR rate of 8.7%, whereas 10.4% was observed in the CLTI group. As expected, the major target limb amputation rate in the claudicants at 0% vs. 5.8% in those with CLTI.

Putting this data into context with other studies is challenging because the SUCCESS study included real-world patients within the full PAD spectrum (RC 1–6) and lesion location (ATK and BTK). The BIOLUX PIII study had a similar-sized patient population and wide inclusion criteria for both PAD presentation and anatomic lesion distribution. 700 patients were enrolled, of which 38% with CLTI and 12% with BTK disease. Lesion length, % of CTOs and incidence of severe calcium were 8.5 cm, 24% and 12% respectively. At 1 year, CD-TLR was similar to SUCCESS at 93.1% despite slightly less complex anatomy [33]. These outcomes are encouraging considering the inclusion of BTK arteries, whereas outcomes on BTK treatment with paclitaxel-coated DCBs are mixed, making it unclear if they benefit over POBA. 4 RCTs based on paclitaxel-coated DCBs failed to succeed with one of them raising a safety concern based on a trend towards a threefold higher amputation at 1 year [13–15, 34]. Only one RCT conducted in a Chinese population with more challenging vascular disease, longer lesions (17 cm) and higher CTO rate (75%) showed benefit of DCB vs. PTA [17].

There is very limited data on DCB treatment of isolated femoropopliteal disease in patients with CLTI to compare with the SUCCESS PTA study. Unlike SUCCESS, with a CLTI population that included 32.6% lesions BTK, The IN.PACT Global study only included CLTI patients with ATK lesions. Within that study, the freedom from CD-TLR was 86.3% in the CLTI group and rate of major target limb amputation was 1.4%. The authors concluded that the treatment of femoropopliteal disease with paclitaxel-coated DCB was safe in patients with CLTI [35]. Results from other ongoing randomized trials comparing limus DEBs to balloon angioplasty balloons (SELUTION4SFA-NCT05132361; SELUTION4BTK-NCT05055297; LIMES-NCT04772300) will help define the role of limus DEBs for treatment in the PAD population.

### Limitations

The SUCCESS PTA Study is a single-arm study, limiting any comparison of the limus DEB with other treatments. Patency was not assessed in this study, which is thought to be a less biased endpoint as compared to freedom from CD-TLR. The registry was intentionally designed to capture contemporary treatment patterns without protocol-driven restrictions. One challenge of an all-comer registry with wide inclusion criteria is depending on the site and operating physician, there were a wide variety of choices available for vessel preparation. Vessel preparation has emerged as instrumental for definitive therapies to exert their full potential. This may be particularly true for DCBs as they miss the scaffolding property which allows stents to overcome elastic recoil. In addition to assigning highest recommendation for the use of DCBs as a definitive therapy in femoropopliteal PAD, a recent Delphi consensus highlighted the merits of multiple vessel prep modalities (particularly specialty balloons) as necessary to pave the ground for patency maintenance [32]. Moreover, the contribution of optimal vessel prep may be even more important in BTK arteries where an absolute lumen loss similar to fem-pop does pay a much higher penalty given the small caliper vessels involved. It may be speculated that a higher and systematic attention to vessel preparation and utilization of vessel preparation specialty devices beyond POBA may play a role in acute and long-term outcomes of this study, as well as past and future trials.

Guidelines for endovascular therapy in claudicant patients are often reserved for patients with disabling symptoms but not often recommended for Rutherford Class 1 or in claudicants with BTK lesions**.** 3.2% of the overall cohort was Rutherford Class (RC) 1, with 4.3% in the claudicant population. There were also patients that underwent BTK treatment with claudication. SUCCESS PTA did not include Rutherford Class as an exclusion criterion. Of the 3.2% of RC 1 patients in the overall cohort, all of those lesions had more than 50% stenosis and were enrolled per investigator discretion based on their site’s standard of care. BTK treatment with SELUTION was reported in 14.1% of claudicant populations (10.1% combined with ATK lesions, 4.0% isolated). It is recommended that treatment of isolated BTK lesions should be avoided [36]. The SUCCESS PTA study is about real-world patients treated per real practice, reflecting different institution or operators’ decisions and offers a reflection point on how guidelines or consensus documents are considered or not considered. The treatment of BTK stenoses in concomitance to an ATK lesion also calls for caution in a claudicant patient. However, the literature suggests that liberal BTK treatment practices still exist in some settings for claudicants as well [37].

## Conclusion

The outcomes from this real-world single-arm study, which enrolled more complex patients, were consistent with similar paclitaxel-coated balloon registries and suggest this limus DEB platform may be an acceptable alternative to paclitaxel-coated balloons, especially in patients with CLTI.

## Supplementary Information

Supplementary Material 1. Supplemental Table 1: Enrolling Centers and Principal Investigators.

## Data Availability

The datasets generated and/or analyzed during the current study contain patient information and are therefore not publicly available due to applicable data protection regulations and ethical restrictions.

## References

[1] Feldman DN, Armstrong EJ, Aronow HD, et al. SCAI consensus guidelines for device selection in femoral-popliteal arterial interventions. Catheter Cardiovasc Interv. 2018;92:124–40. 29691970 10.1002/ccd.27635

[2] Khan MS, Zou F, Khan AR, et al. Meta-analysis comparing endovascular treatment modalities for femoropopliteal peripheral artery disease. Am J Cardiol. 2020;128:181–8. 32650917 10.1016/j.amjcard.2020.05.015

[3] Sachar R, Soga Y, Ansari MM, et al. 1-year results from the RANGER II SFA randomized trial of the ranger drug-coated balloon. JACC Cardiovasc Interv. 2021;14:1123–33. 34016410 10.1016/j.jcin.2021.03.021

[4] Rosenfield K, Jaff MR, White CJ, et al. Trial of a paclitaxel-coated balloon for femoropopliteal artery disease. N Engl J Med. 2015;373:145–53. 26106946 10.1056/NEJMoa1406235

[5] Tepe G, Laird J, Schneider P, et al. Drug-coated balloon versus standard percutaneous transluminal angioplasty for the treatment of superficial femoral and popliteal peripheral artery disease: 12-month results from the IN.PACT SFA randomized trial. Circulation. 2015;131:495–502. 10.1161/CIRCULATIONAHA.114.011004 PMC4323569 25472980

[6] Krishnan P, Faries P, Niazi K, et al. Stellarex drug-coated balloon for treatment of femoropopliteal disease: twelve-month outcomes from the randomized ILLUMENATE pivotal and pharmacokinetic studies. Circulation. 2017;136:1102–13. 28729250 10.1161/CIRCULATIONAHA.117.028893 PMC5598919

[7] Jia X, Zhang J, Zhuang B, et al. Acotec drug-coated balloon catheter: randomized, multicenter, controlled clinical study in femoropopliteal arteries: evidence from the AcoArt I Trial. JACC Cardiovasc Interv. 2016;9:1941–9. 27659572 10.1016/j.jcin.2016.06.055

[8] Mazzolai L, Teixido-Tura G, Lanzi S, et al. 2024 ESC guidelines for the management of peripheral arterial and aortic diseases. Eur Heart J. 2024;45:3538–700. 39210722 10.1093/eurheartj/ehae179

[9] Falkenberg M, James S, Andersson M, et al. Paclitaxel-coated versus uncoated devices for infrainguinal endovascular revascularisation in chronic limb-threatening ischaemia (SWEDEPAD 1): a multicentre, participant-masked, registry-based, randomised controlled trial. Lancet. 2025;406:1103–14. 40902617 10.1016/S0140-6736(25)01585-5

[10] Nordanstig J, James S, Andersson M, et al. Paclitaxel-coated versus uncoated devices for infrainguinal endovascular revascularisation in patients with intermittent claudication (SWEDEPAD 2): a multicentre, participant-masked, registry-based, randomised controlled trial. Lancet. 2025;406:1115–27. 40902614 10.1016/S0140-6736(25)01584-3

[11] Nordanstig J, Hinchliffe R, Lejay A, Behrendt CA, Committee EPADGW, Committee EGS. Editor’s choice - focused update on paclitaxel coated technologies, from the 2024 European Society for Vascular Surgery (ESVS) guidelines on the management of asymptomatic peripheral arterial disease and Intermittent claudication. Eur J Vasc Endovasc Surg. 2026;71:923–927. 10.1016/j.ejvs.2026.04.037 42248619

[12] Liistro F, Weinberg I, Almonacid Popma A, Shishehbor MH, Deckers S, Micari A. Paclitaxel-coated balloons versus percutaneous transluminal angioplasty for infrapopliteal chronic total occlusions: the IN.PACT BTK randomised trial. EuroIntervention. 2022;17:e1445–e1454. 10.4244/EIJ-D-21-00444 PMC9896391 34602386

[13] Mustapha JA, Brodmann M, Geraghty PJ, et al. Drug-coated vs uncoated percutaneous transluminal angioplasty in infrapopliteal arteries: six-month results of the Lutonix BTK trial. J Invasive Cardiol. 2019;31:205–11. 31368893 10.25270/jic/19.3108.205

[14] Zeller T, Beschorner U, Pilger E et al. Paclitaxel-coated balloon in infrapopliteal arteries: 12-month results from the BIOLUX P-II Randomized Trial (BIOTRONIK’S-First in Man study of the Passeo-18 LUX drug releasing PTA Balloon Catheter vs. the uncoated Passeo-18 PTA balloon catheter in subjects requiring revascularization of infrapopliteal arteries). JACC Cardiovasc Interv. 2015;8:1614–22. 10.1016/j.jcin.2015.07.011 26493253

[15] Zeller T, Micari A, Scheinert D, et al. The IN.PACT DEEP clinical drug-coated balloon trial: 5-year outcomes. JACC Cardiovasc Interv. 2020;13:431–443. 10.1016/j.jcin.2019.10.059 32081236

[16] Liistro F, Angioli P, Ventoruzzo G, et al. Randomized controlled trial of acotec drug-eluting balloon versus plain balloon for below-the-knee angioplasty. JACC Cardiovasc Interv. 2020;13:2277–86. 32950416 10.1016/j.jcin.2020.06.045

[17] Jia X, Zhuang B, Wang F, et al. Drug-coated balloon angioplasty compared with uncoated balloons in the treatment of infrapopliteal artery lesions (AcoArt II-BTK). J Endovasc Ther. 2021;28:215–21. 33118432 10.1177/1526602820969681

[18] Linn YL, Choke ETC, Yap CJQ, Tan RY, Patel A, Tang TY. Utility of sirolimus coated balloons in the peripheral vasculature - a review of the current literature. CVIR Endovasc. 2022;5:29. 35748962 10.1186/s42155-022-00308-z PMC9232675

[19] Bohme T, Noory E, Beschorner U, Macharzina R, Zeller T. The SELUTION SLR drug-eluting balloon system for the treatment of symptomatic femoropopliteal lesions. Future Cardiol. 2021;17:257–67. 32815739 10.2217/fca-2020-0085

[20] Tanaka T, Kawakami R, Shiraki T, et al. A pharmacokinetic comparison between three drug delivery devices in porcine coronary arteries. Cardiovasc Revasc Med. 2026;84:36–9. 40947326 10.1016/j.carrev.2025.09.001

[21] Beropoulis E, Avranas K, Rouvi E, Donas KP. Real-world 12-month outcomes with sirolimus-coated balloon angioplasty for complex femoropopliteal disease. J Clin Med. 2025;14:483. 39860489 10.3390/jcm14020483 PMC11766236

[22] Bong TS, Yap CJ, Soon SX, Tang TY. Combination therapy using scoring and sirolimus drug-coated balloons during lower limb endovascular revascularization for chronic limb threatening ischaemia: a case series. SAGE Open Med Case Rep. 2022;10:2050313X221085859. 10.1177/2050313X221085859 PMC8935410 35320985

[23] Franzese M, Pucciarelli A, Spione F, et al. Sirolimus-coated balloon in femoropopliteal steno-occlusive disease: efficacy, safety, and 1-year outcomes. An all-comers registry. J Endovasc Ther. 2025;32:1647–53. 38084379 10.1177/15266028231217657

[24] Iida O, Soga Y, Saito S, et al. A novel sirolimus-coated balloon for the treatment of femoropopliteal lesions: the SELUTION SFA Japan Trial. JACC Cardiovasc Interv. 2024;17:1547–56. 38842992 10.1016/j.jcin.2024.03.029

[25] Tang TY, Yap C, Chan SL, et al. The utility of sirolimus eluting balloons in the setting of chronic limb threatening ischaemia in Asian patients from Singapore - 12 months results of the PRISTINE Registry. Cardiovasc Intervent Radiol. 2024;47:863–74. 38898146 10.1007/s00270-024-03756-3 PMC11239725

[26] Tang TY, Yap C, Soon SXY, et al. World’s first experience treating TASC II C and D tibial occlusive disease using the selution SLR sirolimus-eluting balloon: six-month results from the PRESTIGE Study. J Endovasc Ther. 2021;28:555–66. 33843364 10.1177/15266028211007457 PMC8276341

[27] Zeller T. A novel sustained limus release eluting balloon: 2-year data from the SELUTION SFA Trial. VIVA. Las Vegas, NV. 2019.

[28] Zeller T, Brechtel K, Meyer DR, Noory E, Beschorner U, Albrecht T. Six-month outcomes from the first-in-human, single-arm SELUTION sustained-limus-release drug-eluting balloon trial in femoropopliteal lesions. J Endovasc Ther. 2020;27:683–90. 32666871 10.1177/1526602820941811

[29] Barco S, Engelberger RP, Held U, et al. Sirolimus-coated balloon angioplasty for infrainguinal artery disease. N Engl J Med. 2026;395(6):561-70. 10.1056/NEJMoa2600360 41911022

[30] Teichgraber U, Ingwersen M, Lehmann T, et al. Comparison of sirolimus- vs paclitaxel-coated balloon angioplasty for femoropopliteal artery disease: the SIRONA Randomized noninferiority trial. J Am Coll Cardiol. 2026. 10.1016/j.jacc.2025.12.017. 41706076

[31] Rocha-Singh KJ, Zeller T, Jaff MR. Peripheral arterial calcification: prevalence, mechanism, detection, and clinical implications. Catheter Cardiovasc Interv. 2014;83:E212–20. 24402839 10.1002/ccd.25387 PMC4262070

[32] Korosoglou G, Schmidt A, Lichtenberg M, et al. Global algorithm for the endovascular treatment of chronic femoropopliteal lesions: an interdisciplinary expert opinion statement. JACC Cardiovasc Interv. 2025;18:545–57. 40074516 10.1016/j.jcin.2024.11.038

[33] Tepe G, Zeller T, Moscovic M, et al. Paclitaxel-coated balloon for the treatment of infrainguinal disease: 12-month outcomes in the all-comers cohort of BIOLUX P-III Global Registry. J Endovasc Ther. 2021;27:304–15. 10.1177/1526602819898804 PMC7082893 31989855

[34] Patel A, Irani FG, Pua U, et al. Randomized controlled trial comparing drug-coated balloon angioplasty versus conventional balloon angioplasty for treating below-the-knee arteries in critical limb ischemia: the SINGA-PACLI trial. Radiology. 2021;300:715–24. 34227886 10.1148/radiol.2021204294

[35] Reijnen M, van Wijck I, Zeller T, et al. Outcomes after drug-coated balloon treatment of femoropopliteal lesions in patients with critical limb ischemia: a post hoc analysis From the IN.PACT Global Study. J Endovasc Ther. 2019;26:305–315. 10.1177/1526602819839044 PMC6628633 30931726

[36] Nordanstig J, Behrendt CA, Baumgartner I, et al. Editor’s choice – European Society for Vascular Surgery (ESVS) 2024 clinical practice guidelines on the management of asymptomatic lower limb peripheral arterial disease and intermittent claudication. Eur J Vasc Endovasc Surg. 2024;67:9–96. 37949800 10.1016/j.ejvs.2023.08.067

[37] Mustapha J, Gray W, Martinsen BJ, et al. One-year results of the LIBERTY 360 study: evaluation of acute and midterm clinical outcomes of peripheral endovascular device interventions. J Endovasc Ther. 2019;26:143–54. 30722718 10.1177/1526602819827295 PMC6431778

