# Supplementary material for: Sirolimus-eluting balloon for the treatment of peripheral artery disease: SUCCESS PTA real-world single-arm study
Source: CVIR Endovasc. 2026 Aug 8;9:95. doi: 10.1186/s42155-026-00751-2 (PMC13451607; doi:10.1186/s42155-026-00751-2)
Supplement: Supplementary file 1 [file 42155_2026_751_MOESM1_ESM.docx]

Supplemental Table 1: Enrolling Centers and Principal Investigators

| **Enrolling Centers** | **Location** | **Principal Investigator** |
| --- | --- | --- |
| Alexianer Klinikum Hochsauerland | Arnsberg, Germany | Michael Lichtenberg, MD |
| Attikon University Hospital | Athens, Greece | Elias Brountzos |
| General University Hospital of Patras | Patras, Greece | Konstantinos Katsanos, MD, PhD |
| Universitätsklinikum Essen | Essen, Germany | Christos Rammos, MD |
| Universitätsklinikum Tübingen | Tübingen, Germany | Jörg Schmehl, MD |
| Kantonsspital Aarau | Aarau, Switzerland | Hans Martin Gissler, MD |
| Elisabeth-TweeSteden Ziekenhuis Tilburg | Tilburg, Netherlands | Paul Lohle |
| Guys and St Thomas’ Hospital London | London, United Kingdom | Ashish Patel, MD, PhD |
| Universitätsklinikum Würzburg | Würzburg, Germany | Ralph Kickuth, MD |
| National University Hospital Singapore | Singapore | Rajesh Babu Dharmaraj |
| Athens Medical Center | Athens, Greece | Theodosios Bisdas |
| University Heart Center Freiburg | Freiburg, Germany | Thomas Zeller, MD |
| The Royal Hospital London | London, United Kingdom | Ounali Jaffer |
| GRN Klinik Weinheim | Weinheim, Germany | Grigorios Korosoglou, MD |
| SRH Klinikum Karlsbad | Karlsbad, Germany | Ralph Oberacker MD |
| Frimley Park Hospital | Firmley, United Kingdom | Jeremy D Taylor |
| East Surrey Hospital | Surrey, United Kingdom | Christian Heiss MD |
| Luzerner Kantonsspital | Luzerner, Switzerland | Aljoscha Rastan, MD |
| Hospital Italiano de La Plata | La Plata, Argentina | Julio Andrés Pascua, MD |
| Ludwig Maximilian University  Klinikum Großhadern München | Munich, Germany | Nikolaos Konstantinou |
| University Hospital Leicester | Leicester, United Kingdom | Neghal Kandiyil |
| DIAKO Krankenhaus Flensburg | Flensburg, Germany | Stefan Müller-Hülsbeck, MD |
| Asklepios Kliniken Hamburg | Hamburg, Germany | Joachim Schofer |
| Krankenhaus Buchholz | Buchholz, Germany | Klaus Hertting, MD |
| Universitätsklinikum Heidelberg | Heidelberg, Germany | Dittmar Böckler |
| Fakultná Nemocnica Trnava | Trnava, Slovakia | Lucia Lebdušková |
| Hôpitaux Universitaires de Genève | Geneva, Switzerland | Frédéric Glauser |
